# Contracting Diseases

**Published:** 1876-07

**Authors:** 


### *CONTRACTING DISEASES.*

"I knows what I knows, and I knows what I don't know," said a daft youth to the village miller, who twitted him as to his scanty knowledge. "Indeed," answered the miller, "how is that?"

"Why, I know your pigs are fat, but I don't know whose corn they eat."

Many persons write for the newspapers merely from the store of knowledge they possess, irrespective of what they do not know, and hence a host of crude contributions. The knowledge of many is of a kin in quality, to that of the man who had never seen any land beyond the little island which he had not left from the hour of his birth; he knew that was the only land in the world—because he had seen no other. A fact may come to our knowledge, and we may draw a legitimate conclusion, but additional knowledge may show that it was only part of a fact, and what may be a wise deduction from a part may be a very foolish one when drawn from a whole.

We trust, therefore, that our readers will not conclude us in error from the mere fact that our statements are controverted. The daily and weekly papers very often presume opinions on medical subjects, from the modicum of intelligence which they possess in that direction, whereas if they had known a little more, they would have been ashamed of their immaturity.

### MORNING WALKS MISCHIEVOUS.

Under the head of *Popular Fallacies* we stated, two years ago, that while early rising was commendable, it was better to remain in bed to a late breakfast and then go to work, than to go out of doors for any purpose before breakfast, especially in the summer time, in the west and south-west. It is injurious to the health of anybody in any climate, in any season of the year, to go to any out-door occupation. Some persons may do it with comparative impunity by reason of vigorous health and an active life, but scientific deductions, in common with the ordinary observations of men, are conclusive of the fact, that in all seasons and climates, those who are engaged in out-door occupations, or have to leave their houses early in the morning, ought to eat a regular breakfast before going out; and if that is not practicable, to eat at least a crust of bread or an apple in order to wake up the stomach to an activity which can repel the hurtful influences of cold in winter, and of that *malaria* in summer, which is more or less malignly present about sunrise, wherever a tree flourishes or a blade of grass springs. These are facts known to every educated physician in the world. And yet a contemporary exclaims violently, on the ground of his having been taught from infancy that early rising was healthful,—"Is there no truth?" And to make our assertion appear self-evidently ridiculous he cries out, "Are not birds healthy, and they sing joyously at sunrise?" But to say nothing of the fact that we were speaking as to men and not about brute beasts, a moment's reflection might have suggested, that while it was true that birds had good health and were on the wing early, yet it was not reasonable to suppose that a bird, no more than a man, is inclined to song or mirth on an empty stomach. Besides, as far as our observation has extended among the multitudinous chickens which our mother would always have about her in town and country, not one of them, from the most venerable rooster down to the youngest chicken, ever failed to fly from the tree to the trough. The first instinct of a chick as well as a child is to get a breakfast.

It is not healthy anywhere to get up early and go to work unless something is first eaten. Thousands die every year, and other thousands do more than that, shake with the ague for years in new countries, from failing to eat breakfast before they go out to work. It is useful to observe, that national habits are often the result of their uniformly observed propriety. So what is not healthful in one nation may be in another. Thus it is that the instincts of different nations establish various kinds of food as favorites. Bread is the favorite of the French; maccaroni of the Italian; rice of the Chinese; roast beef of the English; sauerkraut of the German; molasses and pumpkin pie of the New Englander; corn bread of the Kentuckian. The Irishman feasts on his potatoe, while Sawney revels in oatmeal porridge. In the numerous milk-and-water books of travel which annually afflict the reading public, the writers give full frequent evidence of their incapacity, by slighting remarks made of the domestic habits and customs of the people, making their own the standard of perfection, and not taking into judicious consideration the different surroundings of the people among whom they travel.

Literally, incalculable are the good results which would follow a practical attention to these facts. Those who are wise will take no tonics for the spring, will swallow no teas to purify the blood, nor imagine themselves to be about getting sick, because they have not in May as vigorous an appetite as in December, but will at once yield themselves to the guidance of the instincts, and eat not an atom more than they have an inclination for to the end of a joyous spring-time and a summer of glorious health; while those who will eat, who will stimulate the stomach with tonics, and "force" their food, must suffer with drowsiness, depression and distressing lassitude; and while all nature is waking up to gladness and newness of life, they will have no renovation and no well-springs of joyous and exuberant health.

